# Protein Tyrosine Phosphatases in Schizophrenia: A Review of Pathophysiology and Emerging Evidence

**DOI:** 10.7759/cureus.102418

**Published:** 2026-01-27

**Authors:** Karthika Murugesan, Delvy Rebellow, Alousious Kasagga, Aye M Mon, Summayya Anwar

**Affiliations:** 1 Biology, University of North Texas, Denton, USA; 2 Internal Medicine, Malankara Orthodox Syrian Church Medical College, Kochi, IND; 3 Pathology, Peking University, Beijing, CHN; 4 Psychiatry, University of Medicine 1, Yangon, MMR; 5 Biosciences, Commission on Science and Technology for Sustainable Development in the South (COMSATS) University Islamabad, Islamabad, PAK; 6 Research and Development, California Institute of Behavioral Neurosciences & Psychology, Fairfield, USA

**Keywords:** phosphatases, protein tyrosine phosphatases (ptps), ptp1b, ptpn5, ptpra, ptprg, schizophrenia

## Abstract

This article provides a comprehensive review of the molecular and neurodevelopmental mechanisms underlying schizophrenia, with a particular focus on the roles of protein tyrosine phosphatases (PTPs). Schizophrenia is a neurodevelopmental disorder with a complex etiology involving genetic and environmental factors and characterized by diverse clinical symptoms. The review synthesizes recent advances in understanding how dysregulation of specific PTPs, including PTP1B, PTP receptor gamma (PTPRG), PTPN5 (encoding striatal-enriched PTP [STEP]), and PTP receptor type A (PTPRA), contributes to disrupted synaptic signaling, neurotransmitter dysfunction, and neurodevelopmental abnormalities observed in schizophrenia. Key findings include evidence that altered phosphorylation states, impaired myelination, and aberrant modulation of N-methyl-D-aspartate (NMDA) and dopamine receptor function are central to disease pathophysiology. The review also examines the therapeutic potential of targeting PTP1B and other phosphatases, highlighting promising animal model data while emphasizing the need for additional clinical research. Collectively, the article underscores the importance of phosphatase signaling pathways in the pathogenesis and potential treatment of schizophrenia.

## Introduction and background

Schizophrenia is a complex neurodevelopmental disorder with a multifaceted etiology and diverse clinical presentation. It is influenced by genetic variants and a range of environmental factors, including prenatal infections, obstetric complications, and psychosocial stressors [1-3]. Schizophrenia is characterized by a spectrum of symptoms, including positive symptoms such as hallucinations and delusions; negative symptoms such as diminished emotional expression, social withdrawal, and anhedonia; and cognitive impairments that affect daily functioning. The disorder typically manifests in late adolescence or early adulthood and often follows a chronic, relapsing course, imposing significant personal, social, and economic burdens. Early theories of schizophrenia pathophysiology were based on pharmacological studies, particularly those involving antipsychotic agents, and focused on dopaminergic dysfunction [4,5]. Recent advances in neuroimaging, genetics, and molecular biology have revealed a more complex interplay of neurotransmitter systems, neural circuitry, and developmental processes. This review synthesizes current knowledge of schizophrenia etiology, with emphasis on recent findings that advance understanding of the disorder [6]. To explore these developments further, the following section discusses how early pharmacological research shaped foundational hypotheses about schizophrenia.

Early pharmacological studies played a crucial role in shaping our understanding of the disorder, with the antagonism of D2 dopamine receptors being shown to effectively reduce positive symptoms. Conversely, the administration of N-methyl-D-aspartate receptor (NMDAR) antagonists has been found to induce schizophrenia-like symptoms, lending support to both the dopaminergic and glutamatergic hypotheses of the disease [7]. Advances in neuroimaging and postmortem analyses have further elucidated the neuropathological features of schizophrenia. Multiple studies have demonstrated reductions in the expression of ionotropic glutamate receptors across key brain regions, including the prefrontal cortex, thalamus, and temporal lobe, as well as decreased myelin content, atypical neuronal cytoarchitecture, and disruptions in laminar organization [8,9]. These findings collectively point toward disturbed neural development as a central aspect of schizophrenia pathology. Furthermore, contemporary gene expression analyses have revealed abnormalities in myelination [10]. Given these disruptions in neurodevelopment and myelination, researchers have begun to investigate the molecular mechanisms underlying these changes. The following section explores how protein phosphorylation and the activity of specific phosphatases, such as protein tyrosine phosphatase (PTP) 1B (PTP1B), may contribute to these observed abnormalities.

Emerging evidence suggests that dysregulated protein phosphorylation may contribute to the molecular basis of schizophrenia. Abnormal activity of kinases and phosphatases can alter synaptic signaling, glutamate receptor function, and neuronal connectivity, all of which are implicated in the disorder. In this context, PTP1B, a key regulator of phosphorylation-dephosphorylation balance, has gained attention for its potential involvement in neuropsychiatric conditions. Recent studies suggest that aberrant PTP1B signaling may influence NMDAR activity, myelination processes, and synaptic plasticity, pathways that are recognized as disrupted in schizophrenia [11]. This overlap positions PTP1B as a candidate molecular player linking disturbed neurodevelopmental signaling with the clinical and cognitive manifestations of schizophrenia. This review presents an in-depth analysis of the regulatory roles of PTP1B, examines the molecular mechanisms underlying its functions, and highlights recent advances in understanding its involvement in schizophrenia. Beyond PTP1B, other phosphatases such as PTP receptor gamma (PTPRG) also play significant roles in neural development and function. The following section will delve into the classification and functions of these phosphatases, with a particular focus on PTPRG.

## Review

Methodology

A literature search was conducted in PubMed using a combination of keywords and Medical Subject Headings (MeSH) terms, with a focus on schizophrenia and PTPs. Eight studies met the inclusion criteria and were included in the final synthesis. This review covers studies published in English between 2010 and 2025 with full-text availability. Both human and animal studies were included, spanning experimental, observational, systematic, and narrative review designs. Studies published in languages other than English, as well as editorials, letters, and conference abstracts, were excluded because they typically lack sufficient methodological detail and outcome data. Articles older than 15 years were omitted to ensure the synthesis reflects recent scientific knowledge. Materials without peer-reviewed full-text availability, including websites, blogs, posters, and presentations, were excluded due to concerns about reliability and data quality. These criteria were implemented to minimize bias and maintain methodological rigor.

Discussion

Phosphatases are historically classified by substrate specificity into PTPs and protein serine-threonine phosphatases (PSTPs) [12,13]. Within the PTP family, PTPRG is a prominent member of the R5 group, along with PTPRZ, and is categorized as a receptor-like PTP (RPTP) [14,15]. RPTPs feature an extracellular domain (ECD) mediating cell-matrix and cell-cell interactions [16-18] and an intracellular domain (ICD) responsible for enzymatic activity. PTPRG was first identified in the central nervous system (CNS) [16], together with its homolog PTPRZ [19,20], and is now recognized as being ubiquitously expressed. PTPRG mRNA and protein isoforms are found in a diverse range of cell types. In the CNS, these include neuronal and accessory cells such as sensory pyramidal cells, microglia, and Purkinje cells [21-23]. In the CNS, PTPs, including PTPRG, play critical roles in neuronal differentiation and synaptic organization, underpinning processes related to learning and memory [24,25]. This review aims to provide an overview of the emerging roles of PTPRG in schizophrenia. Having established the importance of PTPRG and its related phosphatases, it is helpful to examine other key phosphatases involved in brain function. The following section reviews the role of striatal-enriched PTP (STEP), encoded by the PTPN5 gene, in cognitive processes and its connection to the pathology of schizophrenia.

STEP isoforms: molecular mechanisms and influence on synaptic plasticity

STEP, encoded by the PTPN5 gene, regulates the trafficking of ionotropic glutamate receptors and is essential for cognitive functions, including learning and memory. This functional profile positions PTPN5 as a significant gene in schizophrenia research. Elevated STEP protein levels have been observed in the prefrontal cortex of individuals with Alzheimer’s disease and in several mouse models. Studies by Kurup et al. indicate that these increases result from β-amyloid-induced impairment of proteasomal degradation [26,27]. Given the importance of STEP, exploring how its modulation impacts synaptic plasticity and the broader implications for neuropsychiatric disorders will provide a deeper perspective on the molecular mechanisms at play in schizophrenia.

STEP (also called PTPN5) is an essential modulator of synaptic plasticity and has been increasingly recognized for its involvement in a variety of neuropsychiatric conditions [28]. STEP primarily acts to limit synaptic strengthening, making its precise regulation vital for maintaining healthy cognitive function. Alterations in STEP activity, whether abnormally high or low, are associated with different neurological disorders. Elevated STEP levels have been observed in Alzheimer’s disease, schizophrenia, Parkinson’s disease, and fragile X syndrome [28], whereas reduced activity has been linked to Huntington’s disease, stroke, brain ischemia, and anxiety-related disorders [29]. Given its central role, any dysregulation of STEP can contribute to the cognitive disturbances seen in these diseases.

STEP, an intracellular PTP, exists mainly in two isoforms: STEP46, found in the cytosol, and STEP61, which is membrane-associated [30]. Both forms share a kinase-interacting motif (KIM), which is crucial for recognizing and dephosphorylating substrates within the mitogen-activated protein kinase (MAPK) family [31]. STEP can trigger the internalization of NMDARs and AMPA receptors (AMPARs) and deactivate specific kinases by removing phosphate groups from key regulatory tyrosine residues. The distribution of STEP isoforms varies: STEP61 predominates in the hippocampus, neocortex, lateral amygdala, and spinal cord, while both isoforms are present in the striatum, central amygdala, and optic nerve [32]. STEP46 is primarily involved in suppressing extracellular signal-regulated kinase 1/2 (ERK1/2), while STEP61 binds more strongly to kinases such as Pyk2 and Fyn [33,34]. Research on human postmortem brain tissue from individuals with schizophrenia has shown increased STEP expression in specific cortical regions, likely due to impaired protein ubiquitination. Animal studies support these findings: the administration of noncompetitive NMDAR antagonists, such as PCP or MK-801, elevates STEP levels, and mice lacking STEP exhibit resistance to the behavioral effects of these drugs. Carty et al. also demonstrated that antipsychotic medications can inactivate STEP via protein kinase A (PKA)-mediated phosphorylation, offering insight into potential therapeutic mechanisms for schizophrenia [35]. With this foundation, we can now explore how broader protein regulation systems, such as the ubiquitin-proteasome pathway, intersect with STEP activity.

Carty and colleagues have explored how disruptions in cellular protein regulation can influence brain signaling relevant to schizophrenia [35]. The ubiquitin-proteasome system (UPS), which facilitates the breakdown of proteins, is activated by signals from NMDARs and the entry of calcium into cells [36]. When this system is disturbed, such as by the buildup of β-amyloid, levels of the phosphatase STEP61 can increase, potentially altering synaptic signaling. PCP, a drug that blocks NMDARs and is used to mimic schizophrenia symptoms in research, causes less severe behavioral effects in mice genetically engineered to lack STEP. These mice also exhibit increased NMDARs on the cell surface and higher activity of ERK1/2, suggesting that STEP normally acts to restrict these signaling pathways [37]. Furthermore, antipsychotic medications, including haloperidol, risperidone, and clozapine, appear to increase the phosphorylation of STEP61 through D2 receptor antagonism, which turns off its activity but does not change the total amount of STEP61 [38]. This evidence positions STEP61 as a key regulator connecting protein breakdown, synaptic signaling, and the actions of antipsychotic drugs in schizophrenia.

After considering STEP’s role in cellular protein regulation, attention is next directed to the impact of biological variation on schizophrenia. In this context, sex differences are highlighted as an important factor shaping how the disorder develops and presents across individuals. In schizophrenia, sex differences are well documented. Males typically exhibit earlier onset, poorer premorbid adjustment, less favorable medication responses, and more severe negative symptoms and cognitive deficits than females [39]. The biological mechanisms underlying these disparities remain unclear. Recent evidence suggests that PTPN5 may have a greater impact on schizophrenia risk in males, which could contribute to sex-specific differences in disease onset, symptomatology, and treatment outcomes [26]. Genetic variation in PTPs, especially PTP receptor type A (PTPRA), is increasingly recognized as shaping signaling pathways central to schizophrenia, setting the stage for deeper exploration.

Schizophrenia is widely recognized as a neurodevelopmental disorder involving disruptions in multiple neurotransmitter systems, including those based on dopamine, glutamate, and GABA [40,41]. A recent investigation using whole-exome sequencing in a small, multigenerational family affected by schizophrenia revealed a novel missense variant (p.T577R) in the PTPRA gene, suggesting a possible genetic contribution to disease risk. PTPRA plays an essential role in regulating the phosphorylation state of potassium channels Kv1.1 and Kv1.2, the latter of which is involved in D2 dopamine autoreceptor-mediated dopamine release [42]. In addition, PTPRA modulates the activity of Src family kinases (SFKs), including Src and Fyn, which are critical for the function of NMDARs [43]. Disrupted regulation of these kinases has been associated with NMDAR hypofunction, a prominent feature in schizophrenia [44], with Fyn specifically implicated in NMDAR phosphorylation and trafficking. Altogether, these findings support the role of PTPRA in schizophrenia pathophysiology through its influence on ion channel dynamics, neurotransmitter signaling, and NMDAR regulation [45].

Taken together, these genetic and functional studies highlight PTPRA as a strong candidate gene contributing to schizophrenia risk. Experimental studies demonstrate that loss of Ptpra function leads to several developmental abnormalities. These include reduced peripheral myelination, altered oligodendrocyte maturation, and decreased levels of myelin basic protein (MBP) [46,47]. Further consequences involve disrupted migration of cortical neurons, abnormal orientation of apical dendrites in deep-layer pyramidal neurons, reduced phosphorylation of NMDARs, and impairments in both synaptic plasticity and short-term memory [48,49]. These effects are primarily attributed to the role of RPTPα in modulating the activity of SFKs. PTPRA is located on chromosome 20p13, a region previously associated with susceptibility to schizophrenia [6].

Qin et al. have provided compelling evidence for the role of PTP1B in schizophrenia, demonstrating that mice deficient in LIM domain only 4 (LMO4), an endogenous inhibitor of PTP1B, exhibit distinct schizophrenia-like behaviors [1]. These phenotypes, which can also be induced by the NMDAR antagonist ketamine, are notably prevented by either genetic deletion or pharmacological inhibition of PTP1B [50]. This work underscores the growing interest in PTP1B inhibition as a novel antipsychotic strategy, especially given that some atypical antipsychotics, such as aripiprazole, may function via related mechanisms [11].

The emerging evidence surrounding PTPRA underscores how phosphatase signaling disturbances can shape neurodevelopment and synaptic function in schizophrenia. Extending this concept, recent studies implicate PTP1B in modulating the endocannabinoid (eCB) system, further connecting phosphatase dysregulation to anxiety and schizophrenia-like phenotypes. Cannabis is frequently used for temporary anxiety relief; however, long-term use exacerbates schizophrenia outcomes, particularly in young adults. Evidence suggests a sixfold increase in risk, attributed to alterations in the eCB system [50]. The eCB system, which includes molecules such as 2-arachidonoylglycerol (2-AG) and anandamide, modulates synaptic transmission primarily through presynaptic cannabinoid receptor type 1 (CB1) [1,51].

Emerging therapeutic strategies targeting PTP in schizophrenia

Disruption of this system in animal models, such as through CB1 receptor deletion, results in behavioral deficits that resemble schizophrenia, including impaired cognitive flexibility, reduced prepulse inhibition, and increased anxiety [52]. Studies further demonstrate that mice lacking the PTP1B inhibitor LMO4 in glutamatergic neurons display pronounced early-onset anxiety due to disrupted 2-AG synthesis downstream of metabotropic glutamate receptor 5 (mGluR5) in basolateral amygdala (BLA) [53]. Restoration of eCB signaling, achieved by knocking down PTP1B mRNA or selectively inhibiting PTP1B in the BLA, alleviates anxiety symptoms in these models. Moreover, LMO4 knockout impairs trkB-dependent eCB signaling in prefrontal cortex pyramidal neurons. Genetic removal or pharmacological inhibition of PTP1B, using agents such as trodusquemine, restores trkB activity and reverses associated behavioral abnormalities. Collectively, these findings demonstrate that eCB system dysfunction contributes to schizophrenia-like phenotypes and indicate that targeting PTP1B may provide a promising and reversible therapeutic strategy for adult patients [1].

Building on insights from animal models, we next consider the broader therapeutic potential of PTP1B inhibition, reviewing preclinical evidence across neurological disorders. The therapeutic relevance of PTP1B inhibition extends beyond schizophrenia. Preclinical studies have demonstrated that agents such as trodusquemine and ursolic acid enhance cognitive function, reduce tau hyperphosphorylation, and mitigate amyloid-β toxicity in Alzheimer’s disease models, primarily through the modulation of GSK3β-mediated tau aggregation [53]. Additionally, dampening of microglial-mediated inflammation and decreased brain injury after experimental stroke have been observed with the inhibition of PTP1B. Collectively, these findings position PTP1B as a central signaling node, supporting its inhibition as a promising therapeutic avenue for diverse neurological and psychiatric disorders. Altogether, these findings support the role of PTPRA in the pathophysiology of schizophrenia through its influence on neurotransmitter signaling and regulation of NMDARs.

Limitations

This is a traditional narrative review. We searched PubMed (from 2010 to 2025) and did not perform formal risk-of-bias scoring. While this review highlights essential advances in the understanding of PTPs in schizophrenia, several limitations should be acknowledged. First, much of the current evidence regarding the roles of PTP1B, PTPRG, PTPN5, and PTPRA is derived from animal models or in vitro studies, which may not fully capture the complexity of human schizophrenia. Second, the heterogeneity of schizophrenia’s clinical presentation and underlying pathophysiology poses significant challenges in generalizing findings across patient populations. Third, the interplay between genetic, environmental, and epigenetic factors remains incompletely understood, and the contribution of PTP dysregulation to disease progression may vary among individuals. Finally, while preclinical data on PTP inhibitors are promising, robust clinical trials are necessary to determine the safety, efficacy, and therapeutic relevance of these agents in human patients. Future research should address these gaps to better define the translational potential of targeting PTPs in schizophrenia.

## Conclusions

In summary, growing evidence highlights the critical roles of PTPs in the neurodevelopmental and molecular mechanisms underpinning schizophrenia. Recent studies show that these phosphatases regulate cellular processes, including neuronal differentiation and synaptic connectivity, all of which are essential for brain development and function. Aberrant activity or expression of these enzymes disrupts phosphorylation-dependent signaling cascades, resulting in altered synaptic strength and neural circuitry. Dysregulation of phosphatases such as PTP1B, PTPRG, PTPN5, and PTPRA is increasingly recognized as a key factor associated with the disorder.

This narrative review does not include a formal risk-of-bias assessment. Most of the available evidence is derived from animal models or in vitro experiments, which restricts direct applicability to human populations. While preclinical studies offer valuable insights and suggest novel therapeutic avenues, further research, particularly robust clinical trials, is required to validate these findings and translate them into effective treatments. A deeper understanding of phosphatase signaling in schizophrenia may ultimately inform the development of innovative interventions to improve patient outcomes.
